# Parallel Workflow for High-Throughput (>1,000 Samples/Day) Quantitative Analysis of Human Insulin-Like Growth Factor 1 Using Mass Spectrometric Immunoassay

**DOI:** 10.1371/journal.pone.0092801

**Published:** 2014-03-24

**Authors:** Paul E. Oran, Olgica Trenchevska, Dobrin Nedelkov, Chad R. Borges, Matthew R. Schaab, Douglas S. Rehder, Jason W. Jarvis, Nisha D. Sherma, Luhui Shen, Bryan Krastins, Mary F. Lopez, Dawn C. Schwenke, Peter D. Reaven, Randall W. Nelson

**Affiliations:** 1 Molecular Biomarkers Laboratory, Biodesign Institute, Arizona State University, Tempe, Arizona, United States of America; 2 Center for Innovations in Medicine, Biodesign Institute, Arizona State University, Tempe, Arizona, United States of America; 3 Thermo Fisher Scientific, The Biomarkers Research Initiatives in Mass Spectrometry Center, Cambridge, Massachusetts, United States of America; 4 Phoenix VA Health Care System, Phoenix, Arizona, United States of America; 5 College of Nursing & Health Innovation, Arizona State University, Phoenix, Arizona, United States of America; Swiss Institute of Bioinformatics, Switzerland

## Abstract

Insulin-like growth factor 1 (IGF1) is an important biomarker for the management of growth hormone disorders. Recently there has been rising interest in deploying mass spectrometric (MS) methods of detection for measuring IGF1. However, widespread clinical adoption of any MS-based IGF1 assay will require increased throughput and speed to justify the costs of analyses, and robust industrial platforms that are reproducible across laboratories. Presented here is an MS-based quantitative IGF1 assay with performance rating of >1,000 samples/day, and a capability of quantifying IGF1 point mutations and posttranslational modifications. The throughput of the IGF1 mass spectrometric immunoassay (MSIA) benefited from a simplified sample preparation step, IGF1 immunocapture in a tip format, and high-throughput MALDI-TOF MS analysis. The Limit of Detection and Limit of Quantification of the resulting assay were 1.5 μg/L and 5 μg/L, respectively, with intra- and inter-assay precision CVs of less than 10%, and good linearity and recovery characteristics. The IGF1 MSIA was benchmarked against commercially available IGF1 ELISA via Bland-Altman method comparison test, resulting in a slight positive bias of 16%. The IGF1 MSIA was employed in an optimized parallel workflow utilizing two pipetting robots and MALDI-TOF-MS instruments synced into one-hour phases of sample preparation, extraction and MSIA pipette tip elution, MS data collection, and data processing. Using this workflow, high-throughput IGF1 quantification of 1,054 human samples was achieved in approximately 9 hours. This rate of assaying is a significant improvement over existing MS-based IGF1 assays, and is on par with that of the enzyme-based immunoassays. Furthermore, a mutation was detected in ∼1% of the samples (SNP: rs17884626, creating an A→T substitution at position 67 of the IGF1), demonstrating the capability of IGF1 MSIA to detect point mutations and posttranslational modifications.

## Introduction

Insulin-like growth factor 1 (IGF1) is an important biomarker for the management of growth hormone disorders. IGF1 is produced by the IGF1 gene located on chromosome 12 in humans, and is a critical intermediary involved in cell growth, differentiation, and transformation [1], [2]. Human IGF1 is a 70 amino-acid protein containing three intra-disulfide bonds, with a mass of 7648.7 Da. Serum IGF1 reference values in healthy individuals are 20–600 μg/L [3], [4]. The majority of IGF1 produced acts as an endocrine hormone via secretion from the liver, but the molecule can also serve as a paracrine hormone in certain tissues including cartilaginous cells, and even in autocrine mode as an oncogene [5]–[7]. IGF1 exerts its effects by binding to the IGF1 receptor on target tissues. In blood, 99% of the IGF1 is bound to IGFBPs (Insulin-like growth factor binding proteins), with 80% of circulating IGF1 in a ternary complex consisting of one molecule of IGF1, one molecule of IGFBP3, and one molecule of an acid labile subunit [4], [8]–[10]. As such, the presentation of circulating IGF1 has created the need for methods to disrupt these complexes for accurate IGF1 quantification. For over the past thirty years, IGF1 has been generally quantified using including radioimmunoassay (RIA), immuno-radiometric assay (IRMA), enzyme-linked immunosorbent assay (ELISA), and chemiluminescence [3], [11], [12]. These methods employ various sample preparation steps to disrupt and remove IGFBPs (and thus present free IGF1 for assaying), including low pH, size exclusion chromatography, and acid-ethanol extraction. Some methods also include an addition of IGFBP blocking agent, typically IGF2 [11]. Because of these variations, commercial assays have not provided comparable measurements of serum IGF1 [13].

Since 2001 there has been a rising interest in deploying mass spectrometric methods of detection for measuring IGF1 [14], [15]. Such methods can be categorized as either bottom up (enzymatic digestion followed by analyzing surrogate peptides representative of IGF1) or top-down (no digestion – the intact IGF1 is analyzed directly) and in some instances use immunoprecipitation as a separation step prior to detection. In 2004, our group employed a top-down mass spectrometric immunoassay (MSIA) to quantify IGF1 in human samples [16]. The approach employed a novel sodium dodecyl sulfate (SDS) treatment to disrupt IGFBPs prior to immunoaffinity capture of IGF1, followed by direct detection of intact IGF1 using MALDI-TOF mass spectrometry. Endogenous IGF1 was quantified by introducing a mass-shifted IGF1 analog (internal standard) into samples prior to any processing. The internal standard was present through the entirety of the workflow/analysis, ultimately registering as a mass-resolved signal in the mass spectra that was used for IGF1 signal normalization and quantification (via a working curve method). In 2008, Thevis *et. al.* utilized immunoprecipitation coupled to liquid chromatography/electrospray ionization tandem mass spectrometry (LC/MS) to quantify IGF1 and related analogues [17]. This approach monitored product ions arising from IGF1 terminal peptide fragment dissociations (the molecule was fragmented from its intact form, thus limiting the sequence coverage due to the three remaining intact disulfide bonds). Recently, non-immunoprecipitation MS-based methods for IGF1 quantification have found traction. In 2009, Kay *et. al* implemented an acetonitrile precipitation extraction with a bottom-up LC/MS/MS SRM approach [18]. This approach monitored MS^3^ ions of the n-terminal IGF1 fragment (residues 1–21), provided a dynamic range of 16–2000 μg/L, and had CV's <13%. Solid-phase extraction methods have also been found to work for IGF1 quantification. Bystrom *et. al.* demonstrated top-down IGF1 quantification via solid phase extraction coupled to narrow mass extraction (via Q-TOF) of a single IGF1 isotope in the 7^+^ charge state (m/z 1093.5209) provided excellent analytical metrics (CVs <5.2%, LOD 3.7 μg/L) [19]. Also, Kay *et. al*. further refined their approach, now introducing solid phase extraction (SPE) on the front end of the workflow, and expanded the method to monitor two IGF1 tryptic fragments (1–21, and 38–50) [20]. Each of these approaches has their own merits and taken altogether they clearly show the value of IGF1 quantification with MS detection.

However, beyond providing unambiguous detection of IGF1 with high analytical performance, widespread clinical adoption of any MS-based IGF1 assay will require increased throughput and speed to justify the costs of the analyses (primarily due to unit time and consumables), robust industrial platforms that are reproducible across laboratories (turn-key systems), and the long-term sustainability of supply chains associated with the IGF1 assay (as well as any additional assays). Moreover, and mindful of the ability of mass spectrometry to readily detect molecular variants of a targeted protein [21], the current state of IGF1 mass spectrometric assays could also benefit from enhanced configurations that account for the protein microheterogeneity across populations. Such heterogeneity could feasibly cause currently employed top-down and bottom-up MS approaches to fail, if a patient is phenotypically heterozygous via a single nucleotide polymorphism, or if N- or C-terminal truncations alter the m/z signals that are being monitored in LC/MS. Also, such protein heterogeneity could have yet-undiscovered pathophysiological implications and potential clinical utility.

In this work, we present an MS-based quantitative IGF1 assay that meets all of the above mentioned requirements through achieving these two goals: 1) To rigorously quantify IGF1 in human plasma samples at a rate of >1,000 samples/day, in order to factually benchmark time and economic considerations associated with translating such targeted mass spectrometric assays from research laboratories to clinical deployment, and 2) To accommodate IGF1 heterogeneity discovered from analysis of large populations in order to intelligently design IGF1 mass spectrometric assays that avoid error due to structural variants (or enable study of the variants in alternate disease situations). To the best of our knowledge, neither of these aspects of assay/biomarker translation has been previously put to test and reported on to any substantial degree.

## Materials and Methods

### Human plasma samples

For development of the assay, a bulk quantity (>100 mL) of healthy human EDTA plasma from an individual female donor was purchased from ProMedDX (Norton, MA). One thousand and fifty-four (1,054) human EDTA plasma samples were obtained, with Arizona State University Bioscience IRB approval (Protocol No. 0808003133, Study Title: Oxidative stress in persons with impaired glucose tolerance). The participants signed a written informed consent form that was approved by the IRB. Samples were collected in the following fashion: 20mL of blood was collected into two 10 mL lavender top tubes with EDTA preservative. Tubes were gently inverted several times to prevent the formation of a clot. Samples were then centrifuged at 4°C for 15 minutes at 1300 G (Approx. 3000–4000 rpm). This was completed within 30 minutes of collection time. Using a transfer pipette, plasma was then aliquoted into two 6 mL purple-capped (plasma circled) plastic screw capped cryovials. Samples were frozen within 30 minutes at −80°C after being collected and centrifuged. All samples were then shipped on dry ice and received numbered and without any identifiers. Upon arrival, the samples were thawed and immediately aliquoted into 96 well plates, sealed, and frozen at −80°C.

### Reagents

Polyclonal rabbit anti-human IGF1 affinity purified antibody (Cat. No. PA0362), recombinant human IGF1 (Cat. No. CRI500c), and recombinant human LR3-IGF1 (Cat. No. LRM001) were obtained from Cell Sciences (Canton, MA). Of note, recombinant human IGF1 was the immunogen used to create the polyclonal antibody. Custom MSIA Pipette Tips (Cat. No. 991CUS02), acetonitrile (A954-4), and phosphate buffered saline (PBS, 28372) were obtained from Thermo Fisher Scientific (San Diego, CA). Sinapic acid (Cat. No. 85249), sodium dodecyl sulfate (SDS, Cat. No. 436143), bovine serum albumin (BSA, Cat. No. A4503-10G), trifluoroacetic acid (TFA, Cat. No. 299537), and TWEEN 20 (Cat. No. P7949) were obtained from Sigma-Aldrich (St. Louis, MO). An IGF1 ELISA Kit (Cat. No. RMEE20) was purchased from Biovendor (Asheville, NC). The polypropylene 96-micro titer plates (Cat. No. 651201) were purchased from Greiner Bio-One. Covers used to seal plates (Cat. No. 60941-074) were purchased from VWR.

### Preparation of standards and analytical samples

For the standard curve generation, the IGF1 stock (1 g/L) was serially diluted to 1,500; 1,000; 500; 100; 25; 10; and 5 μg/L, with 100 mM PBS containing 1 g/L BSA (standards buffer). The internal reference standard (LR3-IGF1, 1 g/L) was also serially diluted in standards buffer to a final concentration of 500 μg/L. The human plasma samples and the IGF1 standards were pre-aliquoted into twelve 96-well microplates (40 μL per well) and stored at −80°C prior to use.

### Mass Spectrometric Immunoassay parallel workflow

Steps 1–4 described below were run in a parallel workflow that processed two microplates (192 samples) at-a-time, in an overlapping sequence configuration, resulting in a total processing time of 9 hours for all 12 microplates.

#### Step 1: Sample preparation

On the day of analysis, the first two microplates containing 40 μL of human plasma (or standards) per well were thawed at room temperature (15 minute thaw time). Using an eight-channel electronic pipettor, a 20 μL aliquot of internal reference standard (500 μg/L LR3-IGF1) and a 100 μL aliquot of sample buffer (100 mM PBS w/0.3% (w/v) SDS and 0.1% TWEEN 20) were added to each well, for a total analytical volume of 160 μL in each well. The microplates were then shaken at room temperature for 30 min on an orbital shaker, at 1,000 rpm, to dissociate the IGF1 from the IGFBPs. Total preparation time: <60 minutes.

#### Step 2: Immunoaffinity retrieval and elution

The immunoaffinity retrieval of IGF1 and LR3-IGF1 from the samples was performed using MSIA-Tips derivatized with the IGF1 antibody. Preparation of affinity pipettes were done as previously described for other mass spectrometric immunoassays [22]. Two sets of 96 IGF1 MSIA-tips were mounted on two Multimek 96 automated 96-channel pipettors (Beckman Coulter, Brea, CA) and each set of tips were first rinsed with assay buffer (100 mM PBS w/0.1% TWEEN 20), with 10 cycles (1 cycle consisting of a single aspiration and dispense of a 125 μL volume, ∼3 s), from a single 150 μL buffer aliquot placed in the well of a microplate. Next, the MSIA-Tips were immersed into the wells of the microplates containing the samples, and 100 aspirations and dispense cycles were performed (125 μL volumes each), allowing for simultaneous affinity capture of IGF1 and LR3-IGF1. The MSIA-Tips were then rinsed with assay buffer (100 cycles) from another microplate, and twice with water (10 cycles each) from two more microplates (125 μL volumes aspiration and dispenses, from 150 μL placed in each well). The captured proteins were eluted from the MSIA-Tips by drawing 4 μL of MALDI matrix solution (saturated aqueous solution of sinapic acid, in 33% (v/v) acetonitrile, 0.45% (v/v) trifluoroacetic acid) into the tip and eluting it onto a 96-well format MALDI target. The MALDI targets were air dried for 20 minutes. Total extraction and drying time: <60 minutes.

#### Step 3: MS data collection

MALDI-TOF MS was performed using an Ultraflex III MALDI-TOF mass spectrometer and an Autoflex III MALDI-TOF mass spectrometer (Bruker Daltonics, Billerica, MA). Both instruments were used in linear mode. With the Ultraflex, positive ion, delayed-extraction mode was used with ‘ionsource 1’ at 25.00 kV, ‘ion source 2’ at 23.30 kV, lens at 5.75 kV, 10 ns delayed extraction, deflection signal suppression up to *m/z* 500, and 1 GS/s sample rate. With the Autoflex, positive ion, delayed-extraction mode was used with ‘ionsource 1’ at 20.00 kV, ‘ion source 2’ at 18.50 kV, lens at 8 kV, 130 ns delayed extraction, deflection signal suppression up to *m/z* 4000, and 1 GS/s sample rate. On both instruments, at least six thousand laser-shots were signal averaged for each mass spectrum to ensure good ion counting statistics (approximately 30 seconds per spot). Spectra were externally calibrated with a mixture of 4 proteins, supplied by Bruker (Cat. No. 208241). Total processing time: <60 minutes.

#### Step 4: Data Processing

Individual mass spectra were baseline subtracted (TopHat algorithm) and smoothed (SavitzkyGolay algorithm; width  = 0.2 m/z; cycles  = 1) prior to peak integration using the flexAnalysis 3.0 software (Bruker Daltonics). All peaks representing LR3 IGF1, native IGF1, and IGF1 variants were integrated baseline-to-baseline (using Intrinsic Bioprobes Inc. Zebra 1.0 software) and tabulated in a spreadsheet for quantification. Total processing time: <60 minutes.

### IGF1 Quantification

All samples (plasma and standards) were fortified with a constant amount of the internal reference standard (IRS) (62.5 μg/L LR3 IGF1). For each microplate, a standard curve was generated by plotting the IGF1/LR3-IGF1 peak areas against the concentration of the IGF1 standards, and the data was fitted with a linear trend line using Sigma Plot (Systat Software, San Jose, CA). This standard curve was then utilized to determine the absolute concentration of IGF1 and its variants in the human plasma samples present in the same microplate.

## Results and Discussion

The overall workflow for IGF1 quantification of over one thousand samples, utilizing two pipetting robots and two MALDI-TOF-MS instruments synced into one-hour phases of sample preparation, extraction and MSIA pipette tip elution, MS data collection, and data processing, is shown in **Table 1**. Using this workflow, IGF1 was quantified in 1,054 human samples (frozen plasma – to – data) in approximately 9 hours. This rate of assaying is a significant improvement over existing MS-based IGF1 assays [19], [20], and is on par with that of the enzymatic immunoassays [11]. Although the assay methodology utilized in this work was adopted from our previous work [16], minor modifications were incorporated to achieve the high-throughput analysis while still exploring the unknown molecular landscape of IGF1 protein heterogeneity in large populations should novel variant forms of IGF1 present themselves.

**Table 1:** Parallel workflow for the IGF1 assay.

	8am	9am	10am	11am	12pm	1pm	2pm	3pm	4pm	5pm
Phases (60 minutes each); Number of individuals involved	Plate #									
**Sample Preparation; 1**	**1,2**	**3,4**	**5,6**	**BREAK**	**7,8**	**9,10**	**11,12**			
**Immunoprecipitation and Elution; 1**		**1,2**	**3,4**	**5,6**	**BREAK**	**7,8**	**9,10**	**11,12**		
**MS Data Collection; 2**			**1,2**	**3,4**	**BREAK**	**5,6**	**7,8**	**9,10**	**11,12**	
**Data Processing; 1**				**1,2**	**BREAK**	**3,4**	**5,6**	**7,8**	**9,10**	**11,12**

An important feature of the quantitative IGF1 assay is the addition of the IRS at the beginning of the sample preparation. It is important that the IRS goes through the same processing as does the protein target that is being assayed, to control for possible losses during these processes. Mass-shifted protein analogs may be used as internal standards for MALDI-TOF MS quantification, wherein the internal standard and target protein are captured together using the same antibody [16], [23], [24]. The role of internal standard was fulfilled by Long Arginine 3 IGF1 (LR3-IGF1), an acting molecular variant of IGF1 with a mass of 9,111 Da which contains an arginine substitution at position 3, along with a 13 amino acid n-terminal extension [25]. The mass-shift of +1,462 Da relative to the native IGF1 (7,649 Da) provided a sufficient “open m/z window” to discover unknown variants of IGF1 (should they appear in the mass spectra). Of course, these variants would have to be retrieved by the IGF1 antibody immobilized in the MSIA-Tips. This was made possible by the use of a polyclonal IGF1 antibody, which can capture multiple variants (i.e. LR3-IGF1 and the variants described below) to be unambiguously detected via mass spectrometry. Future studies may precisely determine whether or not the polyclonal antibody has a preference for a specific region or confirmation of the IGF1 molecule to ensure all variants are being captured.

**Figure 1:**
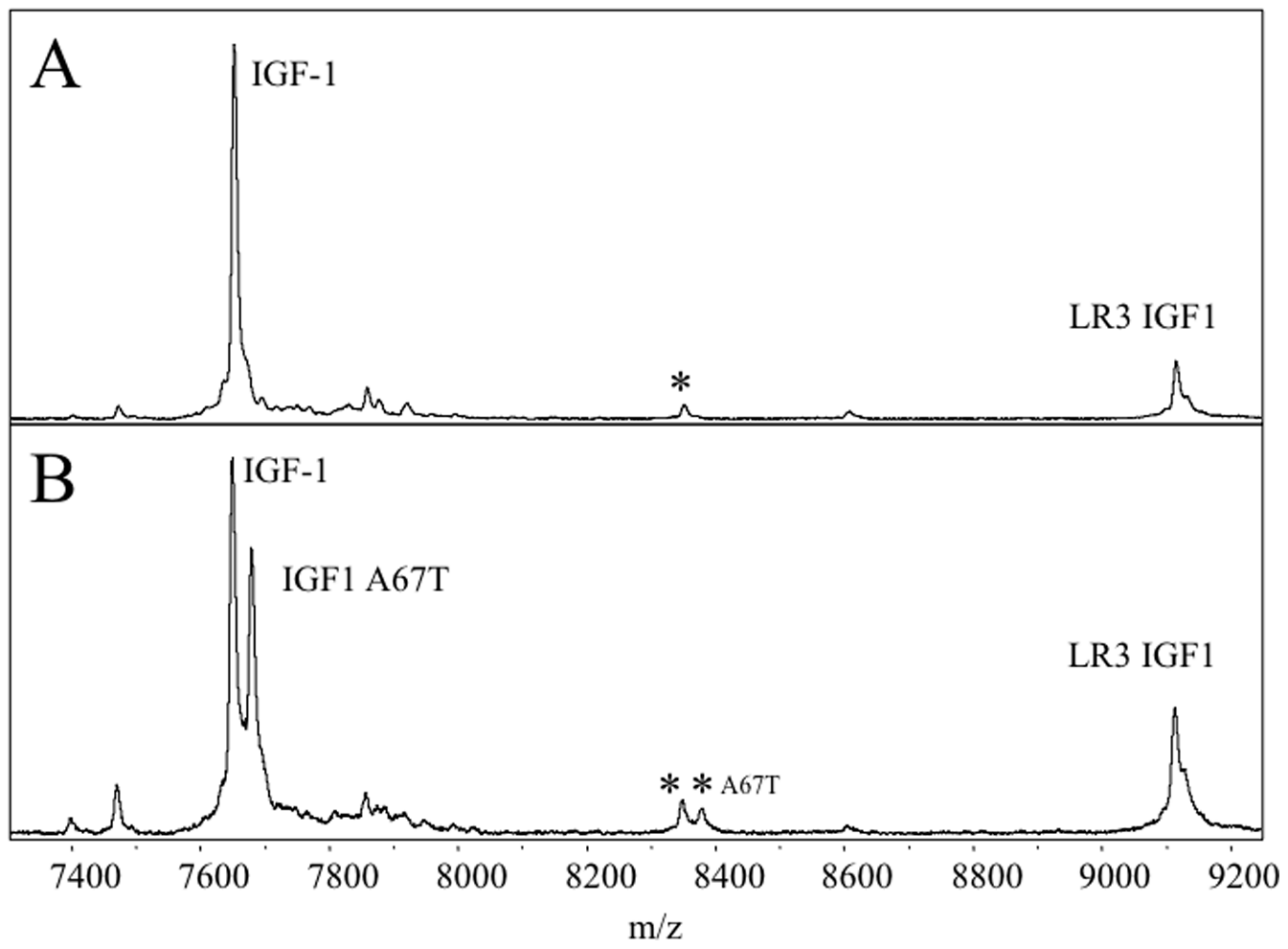
Mass spectra from a normal individual (A) and an individual with a single nucleotide polymorphism (B). m/z values refer to the average mass [M+H]^+^. A) Native IGF1 was detected (observed 7649.99 m/z, theoretical 7649.71) along with a putative glycosylated variant (labeled with *; observed 8346.90 m/z, theoretical unknown). B) Both IGF1 (observed 7649.75 m/z, theoretical 7649.71) and IGF1 A67T (observed 7679.75 m/z, theoretical 7679.71) were detected as well as their respective putative glycosylated variants (observed 8349.35 m/z, theoretical unknown and observed 8379.15 m/z, theoretical unknown).

**Fig. 1** shows two IGF1 MSIA mass spectra representative of the individuals within this study. The first one is that observed from the majority of the samples (“normal IGF1”), while the other represents heterozygous IGF1 detected in approximately 0.9% of the samples, manifesting itself through the appearance of an additional IGF1 signal at +29.7 Da. The mutation giving rise to the heterozygous IGF1 has previously been reported [26] and arises from a single nucleotide polymorphism creating an A→T substitution at position 67 (IGF1 SNP: rs17884626). This mutation is observed primarily in individuals with African descent [26]. The mutation was confirmed by sequencing of the DNA purified from the positive plasma [27].

Another possible variant of IGF1 was identified from the data, at a mass of ∼700 Da higher than IGF1. Interestingly, this signal was also detected in all nine heterozygous samples as a twin-peak accounting for both native and the A→T 67 form of IGF1 (with a mass shift of +29.7 Da). Because of the low relative abundance of this variant, we were unable to determine its exact identity via MS/MS, however, the mass shift potentially corresponds to glycosylation. Further investigations are underway to evaluate this IGF1 variant. Because of the ubiquitous detection of this signal in both homozygous and heterozygous samples, its absence in negative controls, and the use of specific antibody for IGF1 retrieval, it is presumed that this is a glycosylated isoform of IGF1. Lastly, IGF2 and des-Ala IGF2 [28] were also distinctly detected in all mass spectra due to the cross-reactive specificity imparted by the IGF1 antibody. Of note, other top-down IGF1 MS approaches are not configured for detecting variants that are not previously known (e.g. the variants described above).

**Figure 2:**
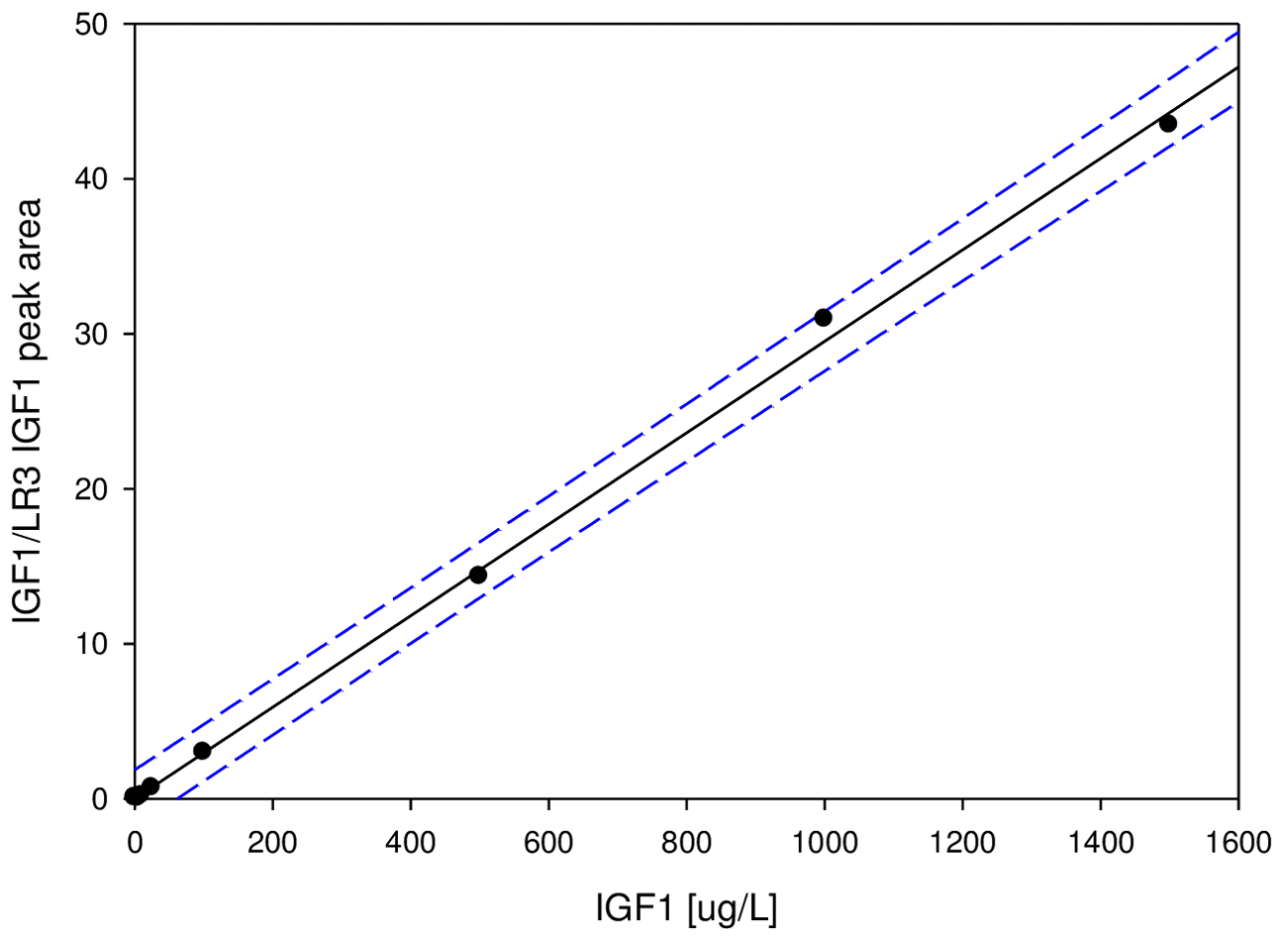
An example of a standard curve for the IGF1 MSIA. Plotted are the peak area ratios of IGF1/LR3-IGF1 over the standards concentrations. Solid line: linear fit with R^2^ = 0.99, SEE =  0.69. Dotted lines: 95% prediction intervals. The average r^2^ for the twelve standard curves was 0.98 and the range was 0.97–0.99.

In all, the IGF1 MSIA standard curves demonstrated excellent linearity (dynamic range of 10^3^) across the normal IGF1 physiological range (5–500 μg/L; average r^2^ = 0.99 for the twelve standard curves), and across higher concentration ranges associated with conditions such as acromegaly (5–1,500 μg/L; average r^2^ = 0.98 for the twelve standard curves). An example of a working curve is shown in **Fig. 2**. The Limit of Detection (defined at S/N = 3) and Limit of Quantification (defined at S/N = 10) were determined to be 1.5 μg/L and 5 μg/L, respectively. The intra-assay precision (within-run) was determined by analyzing six plasma samples, in triplicate, each with a single standard curve. The inter-assay precision (run-to-run) was determined by analyzing two plasma sample three times, on different days, with separate standard curves each time. The results are shown in **Table 2**, and indicate CVs of less than 10%. To determine the linearity of the assay, plasma samples with known IGF1 concentrations were serially diluted, analyzed with the mass spectrometric immunoassay to determine the IGF1 concentrations, and the results compared to those expected (**Table 3**). Spiking recovery experiments were also performed by spiking plasma samples, known to have low IGF1 concentrations, with increasing amounts of recombinant IGF1, followed by analysis with the assay to determine the total IGF1 concentration, and comparison of the results with those expected (**Table 4**).

**Table 2:** Intra-and inter-assay precision.

Intra-assay CVs							Inter-assay CV		
Sample	1	2	3	4	5	6	Sample	1	2
STDEVP:	10.9	11.8	11	19.5	7.7	19.5	STDEVP:	9.31	5.91
MEAN (μg/L):	156	162	140	216	203	204	MEAN (μg/L):	152.6	207.8
CV (%):	6.96	7.28	7.85	9.03	3.79	9.75	CV (%):	6.1	2.85

**Table 3:** Assay linearity.

Sample	Dilution	Observed	Expected	Recovery
		μg/L	μg/L	O/E %
**1**		306		
	2x	146	153	95.2
	4x	72.7	76.60	94.9
	8x	39.1	38.30	102
**2**		239		
	2x	131	120	110
	4x	57.3	59.7	95.9
	8x	34.2	29.9	115

**Table 4:** Spiking recovery.

Sample	Observed	Expected	Recovery
	μg/L	μg/L	O/E%
**1**	112		
	244	259	94.1
	301	339	88.9
	464	419	111
**2**	95.5		
	212	233	91.0
	346	313	111
	414	393	106

**Figure 3:**
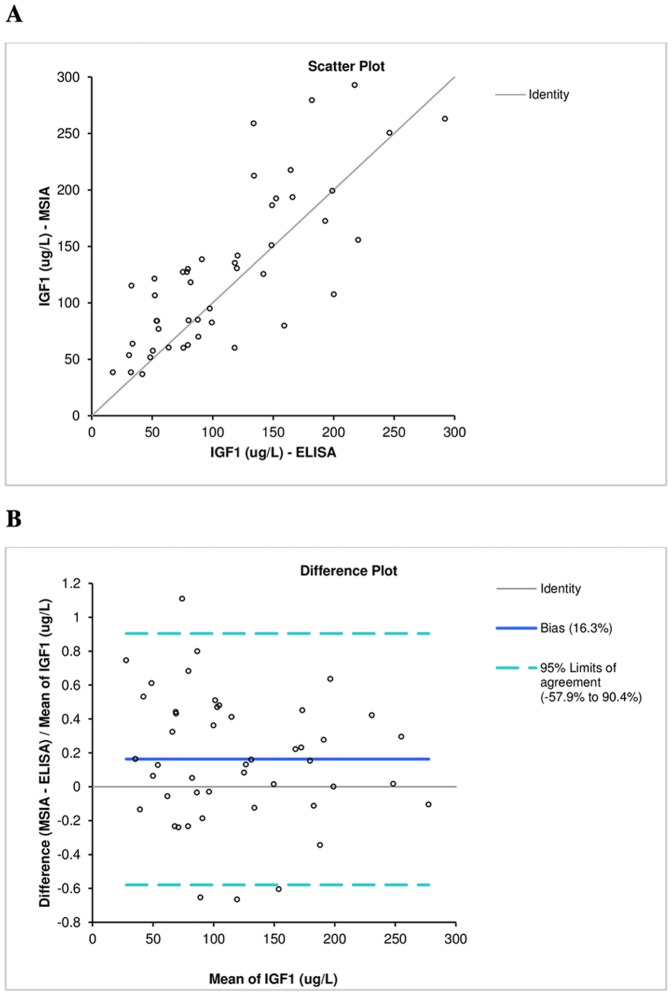
Histogram of IGF1 concentrations determined by MSIA for 1054 EDTA treated human plasma samples.

**Figure 4:**
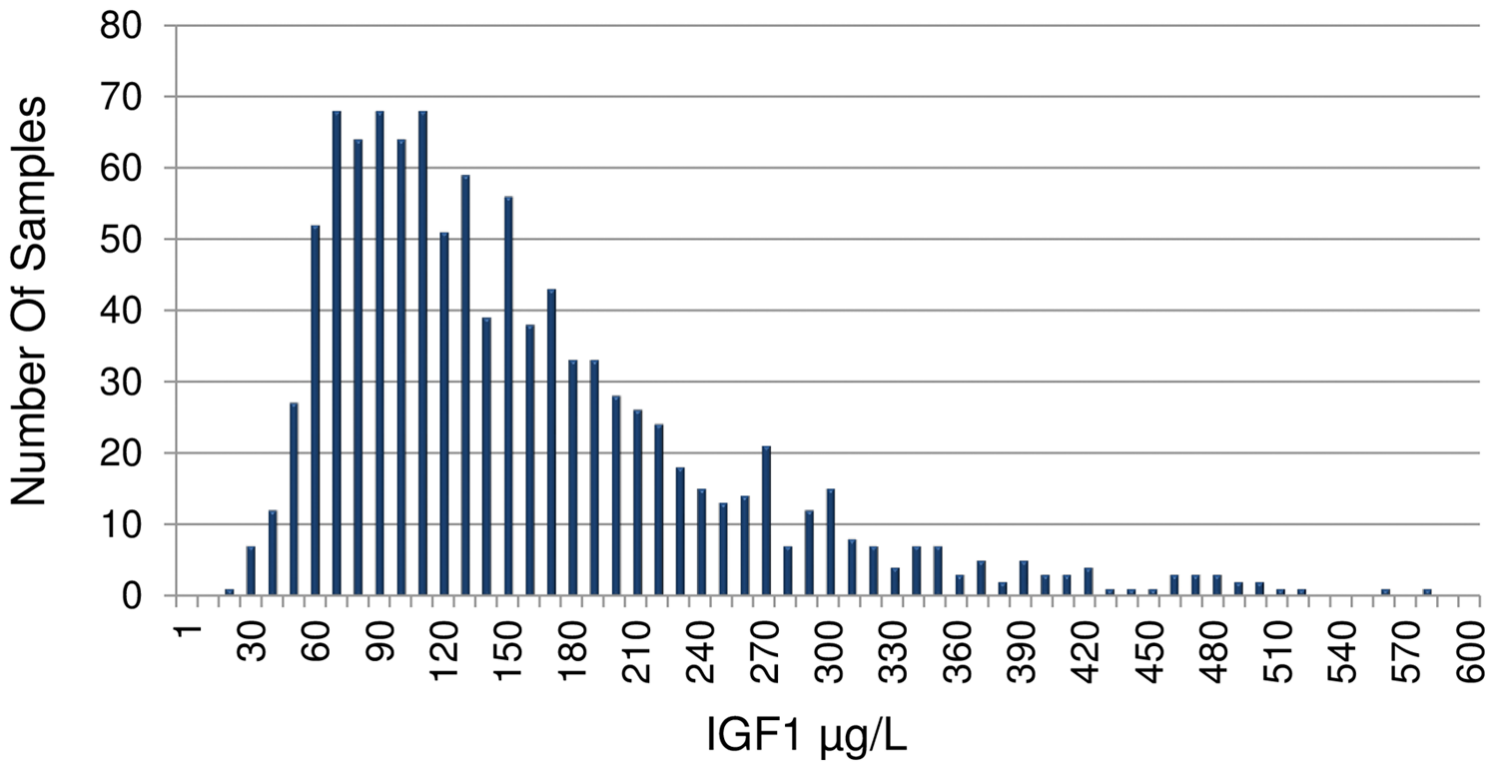
IGF1 methods comparison. A) Scatter plot. B) Difference plot.

For all 1,054 samples, the IGF1 mean concentration and range was determined (using MSIA) to be 152±92 μg/L and range 18–573 μg/L, respectively, which is consistent with the normal reference range of IGF1 [3], [4]. **Fig. 3** shows a histogram presenting the IGF1 concentration range observed in the population. Finally, IGF1 ELISA measurements were performed on 47 representative plasma samples. The IGF1 concentrations determined with the IGF1 MSIA correlated well with those obtained with the IGF1 ELISA, with a Passing Bablock fit [29] of 8.40+1.07x and a Cusum linearity test p>0.1. The Bland-Altman plot [30] shows a slight bias of 16.3% (**Fig. 4**). This is not unusual, and it has been observed in a number of method comparison studies [13], [31].

A second aspect of this study was to evaluate practical clinical viability – as opposed to analytical viability (as just described). Here, topics of interest may include ease-of-operation, time-to-data, throughput and cost-of-data. Regarding the first three topics, we developed and used a simplified sample preparation approach where SDS was added to plasma to disrupt IGF1/IGFBP complexes, after which IGF1 (and internal standard) were retrieved directly from the resulting solution via immunoaffinity capture executed in a pipette tip format. As a result, the transfer of reagents (throughout the overall process) was minimized, and the IGF1 samples never left the original 96-well microplate, thus ensuring more accurate IGF1 measurement. Moreover, the pipette tip extraction format enabled robotic processing (of 96 samples in parallel) of all sample preparation steps – extraction, rinsing and “stamping” of analyte/matrix mixture onto sample plates - leading to mass spectrometric analysis. Data acquisition using MALDI-TOFMS, was rapid, requiring ∼30 s per sample spot to acquire a 5,000 laser shot mass spectrum.

Operating two robot/MS systems realized the analysis of 1,054 clinical samples (plus 96 standard samples) in just over 9 hours (freezer to data analysis), which equates to a rate of just over 30 seconds per sample. The cost for each analysis (i.e., cost-of-data) is broken into unit and fixed costs. Unit costs are those incurred specifically for the analysis, in this case the IGF1 affinity tips, plastic ware and buffers/reagents. Fixed costs are associated with having an analytical platform, keeping it operational and using it, which in this case includes the robot/MS platform, lab space and manpower. The total cost-of-data for MSIA was <$11.25, with unit costs of less than $10 per analysis, and fixed costs of approximately $1.25. Other MS-based approaches typically include more involved sample preparation before analysis and longer times devoted to data acquisition using LC/MS(MS), which lengthens the time-to-data, reduces throughput and increases costs (e.g., a 15-minute LC/MS run at $150/hour [32] equates to ∼$40 in fixed costs on top of any time needed for sample preparation and unit costs). However, the cost-of-data (as well as time-to-data and throughput) demonstrated here is on par, or better than those of current FDA-Approved IGF1 ELISA approaches (e.g., IDS-iSYS).

## Conclusion

The fundamental components of the IGF1 assay (sample preparation, immunoaffinity retrieval, and MALDI-TOF MS) are not novel per se, yet each one contributes significantly to the overall speed and throughput of the assay, positioning it for possible clinical use. Today, the current gold standards in IGF1 assaying are enzymatic immunoassays in the form of ELISAs, which are simple, robust, and affordable. The IGF1 MSIA demonstrated in this work matches the speed, simplicity and cost of the ELISA tests, and offers significant improvements (in these categories) over other reported MS-based IGF1 analyses. Moreover, because MSIA utilizes the very same principle of immunoaffinity capture that is employed in ELISA, but differs in the detection of the analyte binding (using direct detection via high-throughput MALDI-TOFMS instead of ELISAs indirect detection with a secondary antibody), it is able to aid in discovering and distinctly monitoring protein microheterogeneity (e.g. IGF1 A67T) in a single readout. Combined, these characteristics place the mass spectrometric immunoassays at the doorstep of mainstream clinical and diagnostic use.
