# Correction: Parallel Workflow for High-Throughput (>1,000 Samples/Day) Quantitative Analysis of Human Insulin-Like Growth Factor 1 Using Mass Spectrometric Immunoassay

**DOI:** 10.1371/journal.pone.0095812

**Published:** 2014-05-22

**Authors:** The PLOS ONE Staff

Dr. Mary F. Lopez is incorrectly listed as an author on this article. The authors apologize for this error. The correct author list is as follows:

Paul E. Oran, Olgica Trenchevska, Dobrin Nedelkov, Chad R. Borges, Matthew R. Schaab, Douglas S. Rehder, Jason W. Jarvis, Nisha D. Sherma, Luhui Shen, Bryan Krastins, Dawn C. Schwenke, Peter D. Reaven, Randall W. Nelson

The legends for Figures 3 and 4 are incorrectly switched. The publisher apologizes for this error. The correct figure legend pairings can be found below.

**Figure 3:**
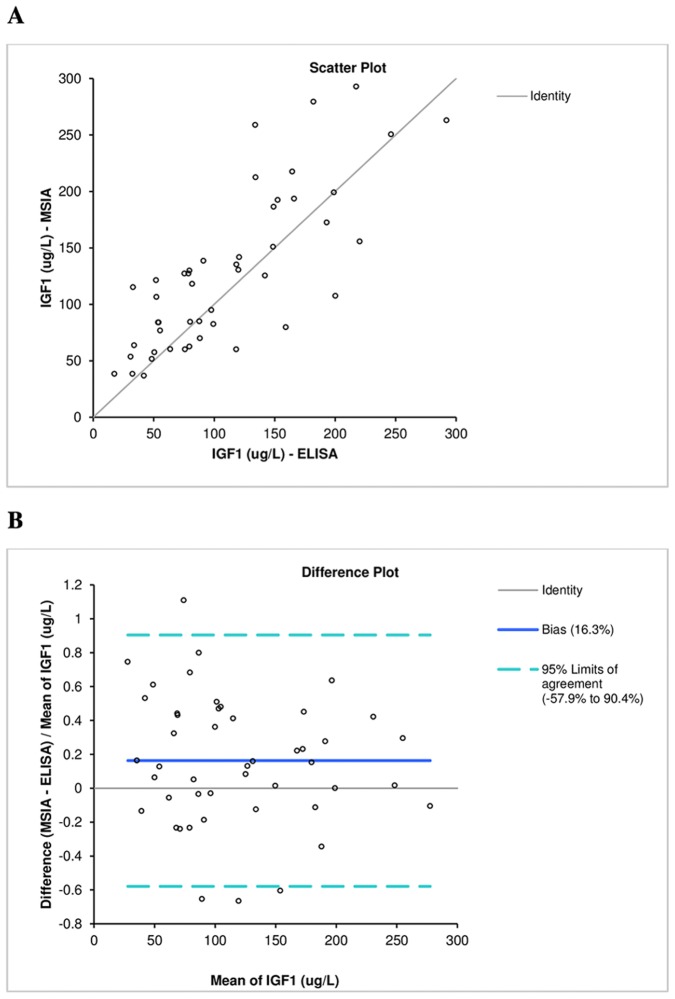
IGF1 methods comparison. A) Scatter plot. B) Difference plot.

**Figure 4:**
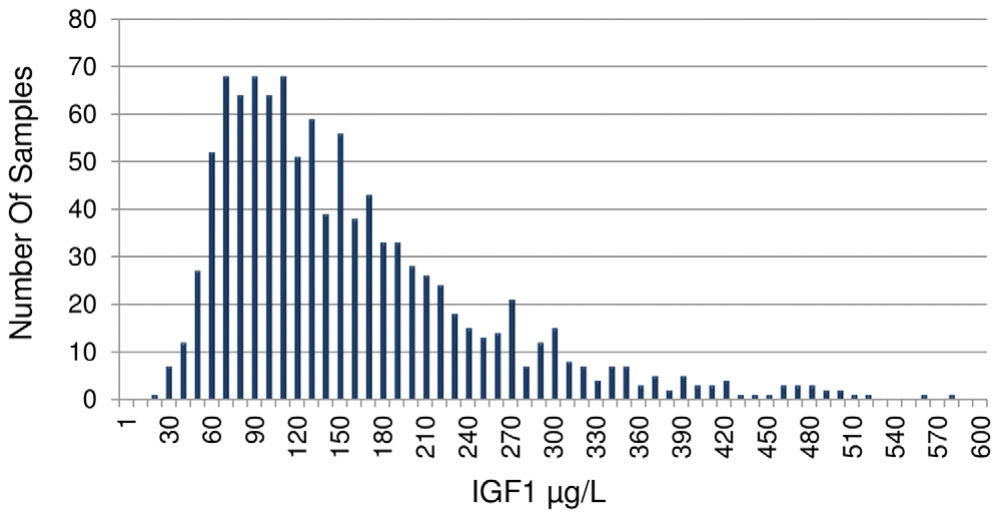
Histogram of IGF1 concentrations determined by MSIA for 1054 EDTA treated human plasma samples.

Additionally, the following sentence should be added to the Acknowledgements section:

“This work was supported in part with resources and of facilities at the Phoenix VA Health Care System. The contents do not represent the views of the Department of Veterans Affairs or the United States Government.”

The complete Acknowledgements read:

“We would also like to express gratitude and acknowledge the following ACT NOW study investigators for their assistance with sample collection: Ralph A. DeFronzo, MD. MaryAnn Banerji, MD, FACP, George A. Bray, MD, Thomas A. Buchanan, MD, Stephen C. Clement, MD, Robert R. Henry, MD, Abbas E. Kitabchi, Ph.D., MD, FACP, FACE, Sunder Mudaliar, MD, Robert E. Ratner, MD, FACP, Frankie B. Stentz, MS, PhD, Nicolas Musi, MD. This work was supported in part with resources and of facilities at the Phoenix VA Health Care System. The contents do not represent the views of the Department of Veterans Affairs or the United States Government.”
